# *Hirsutella sinensis* mycelium regulates autophagy of alveolar macrophages via TLR4/NF-κB signaling pathway

**DOI:** 10.7150/ijms.51654

**Published:** 2021-02-18

**Authors:** Juanhua Fu, Li Lu, Haining Wang, Yayi Hou, Huan Dou

**Affiliations:** 1 The State Key Laboratory of Pharmaceutical Biotechnology, Division of Immunology, Medical School, Nanjing University, Nanjing 210093, China.; 2 Jiangsu Key Laboratory of Molecular Medicine, Nanjing University, Nanjing 210093, China.

**Keywords:** *Hirsutella sinensis* mycelium, pulmonary fibrosis, alveolar macrophage, autophagy, TLR4 signal pathway.

## Abstract

**Background**: *Hirsutella sinensis* mycelium (HSM) has potent anti-pulmonary fibrotic activities and has been proposed as an effective treatment for idiopathic pulmonary fibrosis. Macrophages are the main innate immune cells in the lung tissue, playing key roles in pulmonary fibrosis repair and homeostasis. Excessive macrophage autophagy plays a vital role in pulmonary fibrosis. The protective effect of HSM on macrophages of bleomycin (BLM)-induced pulmonary fibrotic mice remain unclear.

**Methods**: In this study, we collected lung tissue and bronchoalveolar lavage fluid (BALF) samples from pulmonary fibrotic mice. Meanwhile, alveolar macrophages were isolated and murine macrophage RAW264.7 cell line was cultured for further study of HSM autophagy.

**Results:** First, we found that HSM decreased the number of autophagosomes, as well as the levels of LC3B and ATG5, and increased the protein level of P62 during the development of pulmonary fibrosis. Meanwhile, HSM reduced alveolar macrophages infiltration into the BALF and inhibited their accumulation in the fibrotic lung tissue. Flow cytometry analysis showed that HSM administration inhibited the autophagy marker LC3B expression in CD11b^lo^CD11c^hi^ alveolar macrophages in BLM-induced lung fibrosis without affecting CD11b^hi^CD11c^lo^ interstitial macrophages. Transmission electron microscopy and JC-1 staining for mitochondrial membrane potential of alveolar macrophages also verified that the HSM significantly decreased autophagy in the alveolar macrophages of BLM-treated mice. *In vitro*, autophagosomes-lysosome fusion inhibitor chloroquine (CQ) was pre-incubated with RAW264.7 cells, and HSM reduced CQ-induced autophagosomes accumulation. TLR4 signaling inhibitor CLI095 reversed the above effects, suggesting HSM could reduce the cumulation of autophagosomes dependent on TLR4. Furthermore, lipopolysaccharide (LPS)-stimulated TLR4-related autophagy was significantly inhibited by HSM treatment. In addition, the protein expressions of TLR4 and phospho-NF-κB p65 were markedly inhibited in cells treated with HSM.

**Conclusions**: These results indicated that HSM could inhibit the autophagy of alveolar macrophages through TLR4/NF-κB signaling pathway to achieve anti-fibrotic effect.

## Introduction

Idiopathic pulmonary fibrosis (IPF), the archetypal fibrotic lung disease, is a serious disorder with unknown cause and increasing incidence 1-3. The median survival after diagnosis is estimated to be 2-5 years 4, 5. To treat IPF, Pirfenidone and Nintedanib have been included in an update of clinical treatment guidelines in 2015 2. However, these two anti-fibrotic medications are conditionally recommended, and have not been shown to reduce all-cause mortality in sufficiently powered studies 6, 7. A recent conditionally recommended treatment for IPF is anti-acid therapy, proton pump inhibitors or histamine-2-receptor antagonists 8. However, the scientific evidence of their potential beneficial effects on survival remains uncertain 9. Thus, treatment strategies for the pathogenesis of IPF are needed.

In the studies on effective IPF treatments, *Hirsutella sinensis* mycelium (HSM), which is an asexual stage of *Cordyceps sinensis*, has exhibited potent anti-fibrotic activity 10-14. Huang TT *et al.* reported that an ethanol extract of HSM inhibited transforming growth factor-β1 (TGF-β1)-induced differentiation of lung fibroblasts into myofibroblasts and reactive oxygen species production in lung epithelial cells 10. Our laboratory also observed that HSM suppressed mTOR activation stimulated by recombinant TGF-β1 in A549 cells during fibroblast-myofibroblast trans-differentiation 11. These *in vitro* studies were helpful to analyze the anti-fibrosis therapeutic mechanism of HSM, but the exact cause remains unknown. The bleomycin (BLM)-induced infiltration of inflammatory cells into bronchoalveolar lavage fluid (BALF) was found to be reduced by HSM ethanol extract treatment 10. Interestingly, HSM relieved fibrotic damage, accompanied by a decrease in macrophage counts in our previous studies 11, 15. Hence, it is important to examine whether the HSM effect on pulmonary fibrosis is related to macrophages.

Macrophages are the main innate immune cells in the lung tissue 16, and play key roles in the pulmonary fibrosis repair and homeostasis 17. Collagen deposition can be regulated by macrophages, thereby participating in the processing of extracellular matrix 18. Macrophages have been demonstrated to participate in regulating the survival of myofibroblasts 19. Importantly, it has been demonstrated in a rodent BLM model that pulmonary macrophages could be the predominant source of TGF-β, a prominent fibrosis-inducing molecule 20-22. Moreover, patients who developed an accelerated form of fibrotic lung disease were found to have dysregulated alveolar macrophages 23. Some scientists have even suggested that targeting macrophages might have a resolution-promoting role during pulmonary fibrosis 24.

Autophagy is a conserved intracellular degradation pathway, and its disruption compromises homeostasis, which leads to pulmonary diseases 25-27. Increased Light Chain 3 (LC3B) expression in alveolar macrophages was observed in IPF patients, suggesting that excessive macrophage autophagy worsened the disease 28. Similarly, autophagy-related proteins, such as Beclin1 and P62, were expressed in macrophages from the fibrosis areas of paraquat-induced pulmonary fibrosis 29. Autophagosomes accumulated in alveolar macrophages of human silicosis, and promoted macrophage activation to induce the development of fibrosis 30, 31. Taken together, these data suggested that macrophage autophagy might play a vital role in pulmonary fibrosis.

The current study aimed to investigate the relationship between HSM alleviation of pulmonary fibrosis and macrophage autophagy, as well as the underlying molecular mechanism. The results showed that HSM could effectively inhibit excessive macrophage autophagy in BLM-induced pulmonary fibrosis mouse model, and the TLR4/NF-κB signaling pathway was required for HSM effect on macrophage autophagy.

## Materials and Methods

### Chemicals and reagents

HSM was obtained from Nanjing Zhongke Group (Nanjing, China), and the HSM solution was prepared as previously described 11. BLM was purchased from Hisun Pharmaceutical Co., Ltd. (Zhejiang, China). Chloroquine (CQ), lipopolysaccharide (LPS), collagenase I and collagenase IV were obtained from Sigma (St. Louis, MO, USA). DNase I was purchased from Roche (Switzerland, UK). Dulbecco's Modified Eagle's Medium (DMEM) and Roswell Park Memorial Institute (RPMI) 1640 medium were obtained from Hyclone Laboratories (South Logan, UT, USA). Fetal bovine serum (FBS) was purchased from Gibco (Grand Island, NY, USA). JC-1 was obtained from Fcmacs (Nanjing, China). TLR4 signaling inhibitor CLI095 was purchased from Invitrogen (Carlsbad, CA, USA). The antibodies used in this study were rabbit anti-GAPDH (Good HERE, Hefei, China), rabbit anti-LC3B (Cell Signaling Technology, MA, USA), rabbit anti-P62 (Cell Signaling Technology, MA, USA), rabbit anti-ATG5 (Cell Signaling Technology, MA, USA), rabbit anti-NF-κB p65 (Cell Signaling Technology, MA, USA), rabbit anti-phospho-NF-κB p65 (Cell Signaling Technology, MA, USA), rabbit anti-ERK (Cell Signaling Technology, MA, USA), rabbit anti-phospho-ERK (Cell Signaling Technology, MA, USA), rabbit anti-IRF 3 (Cell Signaling Technology, MA, USA), rabbit anti-phospho-IRF 3 (Cell Signaling Technology, MA, USA), PerCP anti-mouse CD45 (clone number: 30-F11) (Biolegend, CA, USA), APC anti-mouse CD11c (clone number: N418) (eBioscience, CA, USA), FITC rat anti-CD11b (clone number: M1/70) (BD Biosciences, CA, USA), PE anti-rabbit LC3B (clone number: D11) (Cell Signaling Technology, MA, USA), goat anti-rabbit IgG (H+L) (Fcmacs, Nanjing, China) and goat anti-rabbit IgG (Fcmacs, Nanjing, China).

### Mouse model of pulmonary fibrosis

Specific pathogen-free (SPF) male C57BL/6 mice (aged 7-8- weeks, weighting 20 ± 2 g) were purchased from GemPharmatech [SCXK (SU) 2018-0008, Nanjing, China]. All animal experiments were strictly in accordance with the National Institutes of Health (NIH) Guidelines for the Care and Use of Laboratory Animals, and approved by the Institutional Animal Care and Use Committee of Nanjing University (Nanjing, China). All mice were housed in SPF condition with a 12:12 h light-dark cycle, and received water and food ad libitum. Mice were acclimated for at least a week before use.

**Figure 1:**
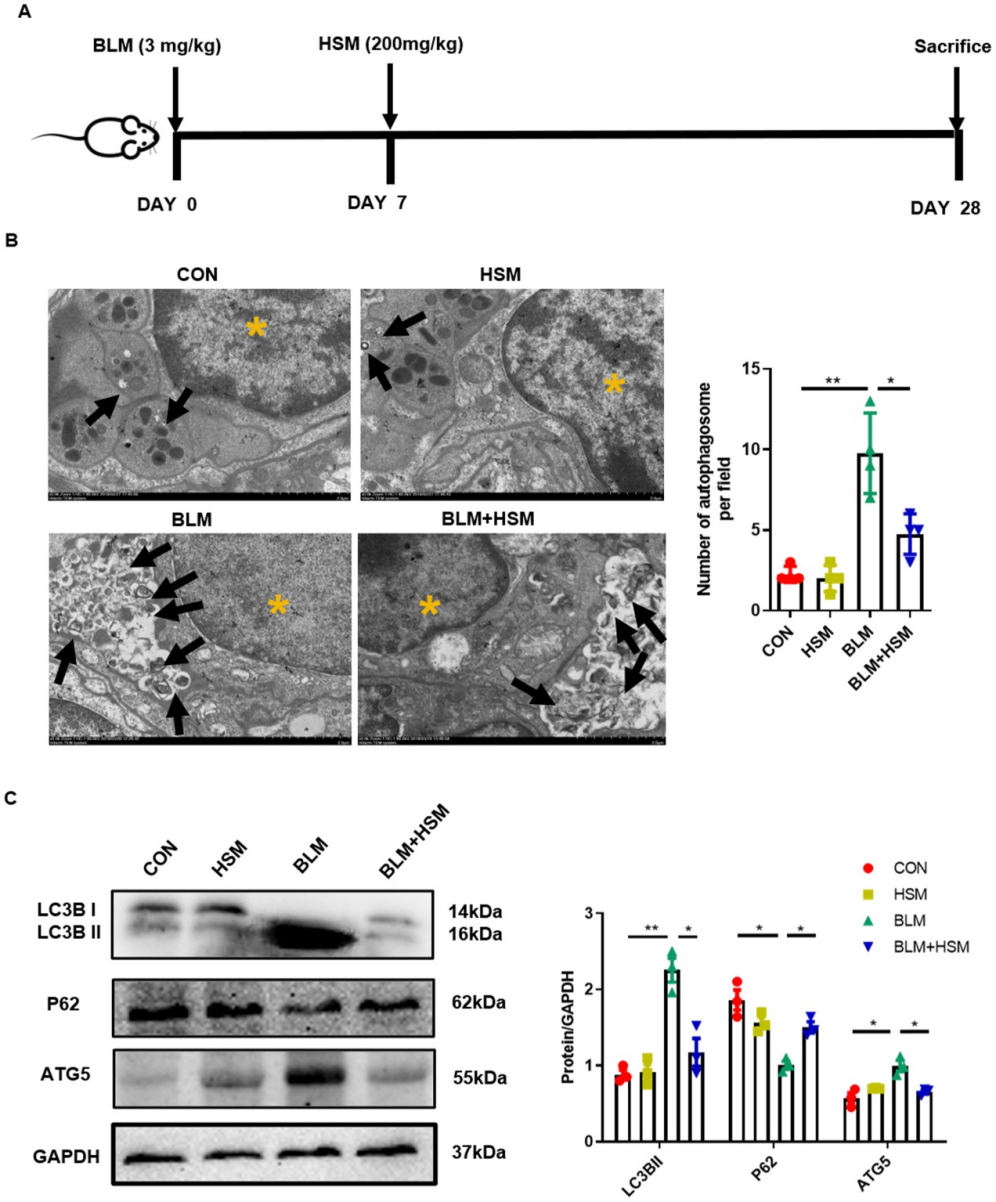
** HSM down-regulates excessive autophagy in BLM-induced pulmonary fibrosis mice.** (A) *In vivo* experimental design: Mice were intratracheally administered with BLM (3 mg/kg in 100 μl saline) on day 0, and HSM (200 mg/kg in 500 μl saline) was orally administered after the BLM administration from day 7 to day 27. On day 28, the mice were sacrificed and the corresponding samples were collected. (B) Transmission electron microscopy image of mouse lung tissue. Black arrow: autophagosome; yellow asterisk: cell nucleus; scale bar: 2 μm. The black bars represent the mean ± SEM values between the treated groups analyzed, n=4. (C) Analysis of the changes of LC3B, P62 and ATG5 proteins in mice of each group by Western blotting, n=3. Data are presented as means ± SEM of at least three separate experiments. *P < 0.05, **P < 0.01.

The experimental process is shown in Figure 1A. A total of 40 male C57BL/6 mice were randomly divided into four groups: (i) CON group, (ii) HSM group, (iii) BLM group, (iv) BLM+HSM (200 mg/kg) group. The BLM stock solution was prepared by dissolving the compound in sterile saline and storing small aliquots at 4 °C. BLM (3 mg/kg) was intratracheally administered to induce pulmonary fibrosis on day 0. The CON and HSM groups were treated with the equivalent volume of vehicle (saline). From day 7 to 27 after the BLM administration, HSM diluted in 500μl saline or vehicle control (saline) was administered by oral gavage once daily. On day 28, all the mice were sacrificed.

### Sample collection

After the mice were sacrificed, the lungs and part of the trachea were harvested. The anterior lobe of the right lung was inflated, fixed in 4% paraformaldehyde for 24 h, and then paraffin embedded. The middle lobe of the right lung was collected for transmission electron microscopy (TEM) observation. After the ligation, 1 mL of PBS was injected into the lungs through the catheter, the lungs were washed five times and lavage fluids were collected for subsequent detection. After the lavage, lower lobe of left lung tissue was removed for flow cytometry analysis, gene and protein testing.

### Isolation of alveolar macrophages and transmission electron microscopy observation

BALF was centrifuged (300 g, 5 min, 4 °C) to obtain a pellet, the pellet was lysed with erythrocyte lysate, then resuspended in RPMI-1640 medium (containing 10% FBS and 1% penicillin streptomycin). Cells (1×10^6^ cells/well) were seeded in 6-well plates overnight. On the second day, the non-adherent cells were discarded, and 10 ml of EDTA-2Na was added to detach the adherent cells. After 30 min, the adherent cells were recovered, the supernatant was centrifuged, and washed three times with RPMI 1640 medium to obtain purified alveolar macrophages.

The purified alveolar macrophages and the lung tissue were fixed with phosphate buffer solution at pH=7.4 containing 2% paraformaldehyde and 2.5% glutaraldehyde. Alveolar macrophages were sliced at 70-80 nm with a diamond knife, and the slices were placed on a copper grid. Tissue samples were then fixed with 1% phosphate-buffered osmium tetroxide, embedded in Spurr's resin, and then sliced. After sectioning, the slices were stained with 1% uranyl acetate and 0.2% lead citrate. The slices were sent to Nanjing Google Bio Company (Nanjing, China) for imaging and observing the number of autophagosomes.

### Cell culture and viability analysis

The murine monocyte/macrophage RAW264.7 cell line was cultured in a culture flask containing 10% FBS in DMEM high glucose medium and placed in an incubator at 37°C and 5% CO2 saturated humidity. In the *in vitro* experiment, RAW cells were pre-treated with CQ (20 μM) for 2h, followed by 16 μg/ml HSM treatment for 16 h. Similarly, LPS (0.1 μg/ml) and CLI095 (100 μg/ml) were used to treat cells for 2 h before using HSM (16 μg/ml and 160 μg/ml).

RAW264.7 cells (2 x 10^4^ cells/well) were seeded in 96-well plates to assess cell viability. After overnight incubation, cells were incubated with various concentrations of HSM (6.25-1600 μg/ml) for 48 h. Subsequently, 10 μl of CCK-8 solution was added to each well and incubated for an additional 2 h. The absorbance at 450 nm was measured by a microplate reader (Bio Tek, Winooski, VT, USA). The effect of HSM on the viability of RAW264.7 cells was shown as ratios of the treated group to control group, and repeated for three times.

### Western blotting

Lung tissues and cells were lysed in ice-cold RIPA lysis buffer containing protease and phosphatase inhibitors. The supernatants were collected by centrifugation and quantitated through BCA protein assay kit (Beyotime Institute of Biotechnology). An equivalent amount of 30 μg protein was separated by SDS-PAGE, transferred to a 0.22 μm PVDF membrane (Millipore Co, Bedford, MA, USA), and blocked in 5% BSA for 2 h at room temperature. The PVDF membrane was then incubated with the primary antibody overnight at 4 °C followed by incubation with horseradish peroxidase-conjugated goat anti-rabbit IgG secondary antibodies for 2 h in the dark. An ECL kit (Supersignal^TM^ West Pico PLUS, Thermo, USA) was used to detect the protein bands, and the grey level of proteins were calculated by the Image J software (National Institutes of Health, Bethesda, MD, USA). We diluted the antibodies as follows: rabbit anti-GAPDH (1:1000), rabbit anti-LC3B (1:1000), rabbit anti-P62 (1:1000), rabbit anti-ATG5 (1:1000), rabbit anti-NF-κB p65 (1:1000), rabbit anti-phospho-NF-κB p65 (1:1000), rabbit anti-ERK p65 (1:1000), rabbit anti-phospho-ERK (1:1000), rabbit anti-IRF 3 (1:1000), rabbit anti-phospho-IRF 3 (1:1000) and goat anti-rabbit IgG (1:4000). In the experiment, GAPDH was used as a protein loading control.

### Extraction of total RNA and quantitative real-time PCR

Total RNA was isolated from RAW264.7 cells or lung tissues using the Trizol reagent (Invitrogen, USA). Thereafter, 1 mg RNA was reverse-transcribed into cDNA by HiScript® II Q PT SuperMix for qPCR kit (Vazyme Biotech, Nanjing, China). Next, qRT-PCR was performed on the BIOER Line Gene 9640 (Hangzhou, China). The relative expression of each gene was calculated by the 2^-ΔΔCt^ method, repeated ≥3 times. The mRNA levels were normalized to that of GAPDH. The primers were synthesized by Springen (Nanjing, China). The 5′—3′ primer sequences were as follows:

m-LC3B: forward primer TGACTCACCTTGTGGTCCTAA, reverse primer CTTCCCAGAATCCAGTCTTTCC;

m-GAPDH: forward primer AGGTCGGTGTGAACGGATTTG, reverse primer TGTAGACCATGTAGTTGAGGTCA.

### Flow cytometric analysis

ACK buffer was used to lyse the excess red blood cells in BALF. For the lung tissue, pulmonary lobes were mechanically disrupted in gentleMACS^TM^ Dissociator (Miltenyi Biotec, Germany), digested in RPMI 1640 medium containing collagenase I (3 mg/ml), collagenase IV (3 mg/ml) and DNase I (2.5 μl) and filtered through a 70 μm cell strainer to remove major tissue fragments. Single-cell suspensions were then fixed with 4% paraformaldehyde and stained with Percp-conjugated CD45, APC-conjugated CD11c and FITC-conjugated CD11b antibodies for 30 min at 4 °C in the dark. After the cells were fixed and permeabilized, the intracellular antibody LC3B-PE was stained. Accuri C6 (BD Biosciences, CA, USA) was used to detect fluorescence, and FlowJo software (TreeStar, Ashland, OR, USA) was applied for data analysis.

### Immunofluorescence

RAW264.7 cells were cultured on a glass coverslip. After various treatments, the cells were fixed with 4% paraformaldehyde at room temperature for 30 min, and 0.1% Triton X-100 at room temperature for 15 min to increase the permeability of the cell membrane. After blocking with 3% bovine serum albumin at room temperature for 60 min, the cells were incubated with rabbit anti-LC3B antibody at 4°C overnight followed by incubation with the goat anti-rabbit IgG (H+L) secondary antibody for 2 h at room temperature in the dark. The cells were stained with DAPI (Fcmacs, Nanjing, China), and finally the coverslip was fixed with neutral glue. Then the coverslip was observed under a FV10i confocal microscope (OLYMPUS, Japan).

### Statistical analysis

All data were expressed as mean ± S.E.M. Statistical analysis was performed using Prism8 software, and one-way Analysis of Variance (ANOVA) was used for comparison between groups. A *p-value* <0.05 was considered statistically significant. The experimental results were repeated three more times, and each data point represents the mean of at least three samples.

## Results

### HSM down-regulates excessive autophagy in BLM-induced pulmonary fibrosis mice

As shown in Fig. 1A, we used BLM-induced pulmonary fibrosis model, and subsequently HSM was used for drug therapy (for details, please refer to the Materials and Methods section). In our previous studies, HSM was found to mitigate lung injury and fibrosis 11, 32, which prompted us to explore the underlying mechanism. Autophagy is involved in the pathological progression of IPF 33, 34. Autophagosome formation and autophagic flux were reported to significantly increase in patients with lung fibrosis as well as BLM-induced pulmonary fibrosis animals 35, 36. We first applied TEM to observe the autophagosome inside the lung tissue slices, as a golden standard 37. We quantified the TEM sections and found that the number of autophagosomes were significantly increased in the BLM-induced fibrosis lung tissue, compared to uninjured saline control lung. After the HSM treatment, autophagosome formation per tissue area was significantly reduced compared with the BLM model group (Fig. 1B). LC3B hydrolyzes into cytosolic LC3B-I and lipid autophagosome membrane-bound LC3B-II. The conversion of LC3B-I to LC3B-II is an essential event for autophagosome formation. An increase in LC3B-II level is, therefore, a marker of activated autophagy 38-40. As an LC3-interacting protein, P62 begins to degrade followed by the progression of autophagy 41. ATG5 is another key autophagy protein required for conjugation of the ubiquitin-like protein LC3 to the phagophore 42. Next, LC3B, P62 and ATG5 in lung tissue extracts were analyzed by western blot. LC3B-II level was remarkably increased at 28 days post BLM administration compared to saline control, while a decrease in P62 level was also observed (Fig. 1C). Hence, the autophagy level in the BLM model group was higher, consistent with a previous report 35. Compared to tissue from the BLM group, significantly lower level of LC3B-II was observed in the BLM+HSM group, and the expression level of P62 was also up-regulated. Moreover, ATG5 expression showed a similar variation as LC3B-II between different groups. These results indicated that HSM regulates autophagic activity in the pathogenesis of pulmonary fibrosis.

### HSM reduces the number of alveolar macrophages in the fibrotic lung

**Figure 2:**
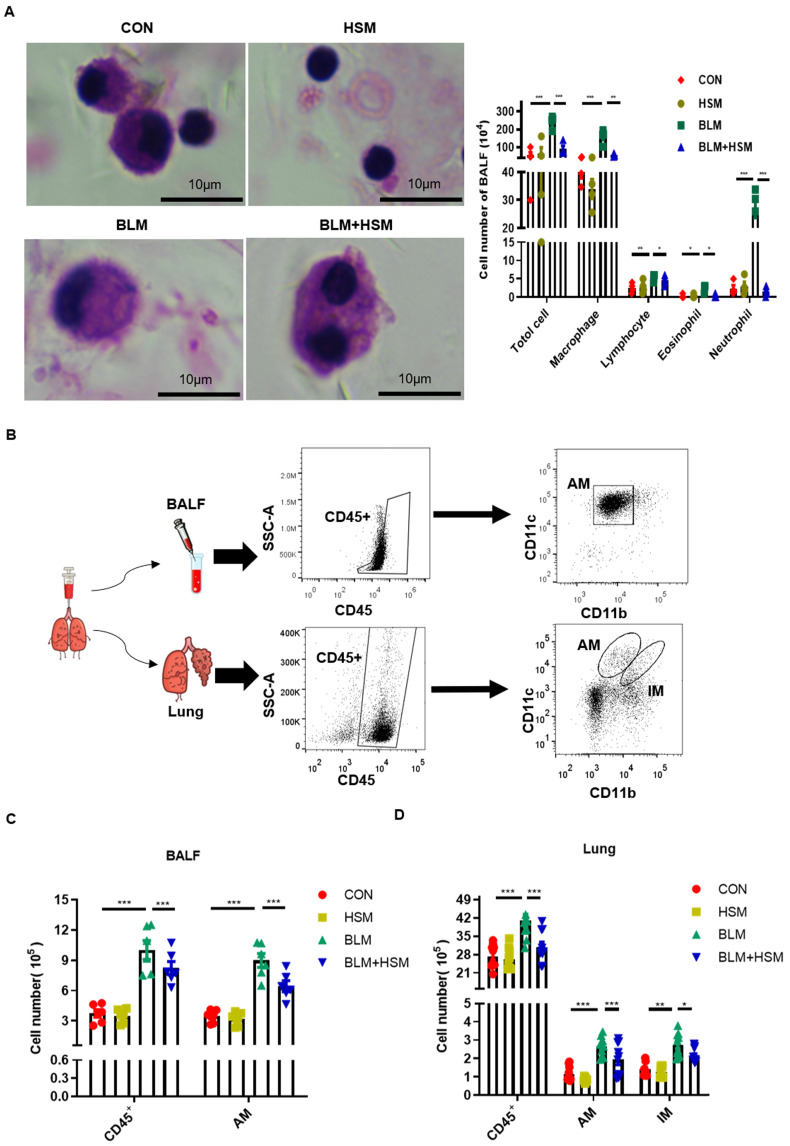
** HSM reduces the number of alveolar macrophages in the fibrotic lung.** (A) BALF was centrifuged and coated to count the changes of total cells, macrophages, lymphocytes, eosinophils and neutrophils. The CON, HSM, BLM and BLM+HSM mainly display representative morphological images of macrophages, lymphocytes, eosinophils and neutrophils. Scale: 10 μm, magnification: x200, n=4. (B) Gating strategy mainly used to identify macrophages in BALF and lung tissue of mice. The differential expression of CD11b and CD11c was used to distinguish pulmonary macrophages. (C) Effect of HSM on the number of total cells, CD45^+^ cells and alveolar macrophages in BALF, n=6. (D) Effect of HSM on the number of total cells, CD45^+^ cells, alveolar macrophages and interstitial macrophages in lung tissue, n=10. Data are presented as means ± SEM of at least three separate experiments. *P < 0.05, **P < 0.01, ***P < 0.001.

Since inflammatory cells in BALF interact with lung tissue cells to play an important role in pulmonary fibrosis, we attempted to identify whether these cells were associated with HSM regulation of autophagic activity in BLM-induced mice 43, 44. Therefore, H&E staining was used to examine the changes in the numbers of various inflammatory cells in BALF. Based on the morphology of various cells, administration of BLM caused extensive infiltration of inflammatory cells, as shown by significant increases in total cells, macrophages, lymphocytes, eosinophils and neutrophils (Fig. 2A). As expected, HSM reversed the total cell and leukocyte accumulation in BALF of BLM-treated mice (Fig. 2A). Among these immune cells, macrophages attracted our attention because of their huge advantages in quantity. Next, the effect of HSM on macrophages was further analyzed by flow cytometry.

Pulmonary macrophages are mainly divided into two groups: alveolar macrophages (AMs) located in the airway space 45, and interstitial macrophages (IMs) residing in the lung parenchyma 45, 46. AMs and IMs can be distinguished by the differential expression of their surface markers: integrin CD11b and CD11c. AMs express low levels of CD11b and high levels of CD11c, while IMs express high levels of CD11b and low levels of CD11c 47. Therefore, flow cytometry was applied to detect cell surface proteins as previously described, and the specific experimental process is shown in Fig. 2B 48. The number of total CD45^+^ leukocytes in the BALF of BLM-treated mice was higher than the control mice. In contrast, the treatment with HSM reduced the number of infiltrated CD45^+^ cells (Fig. 2C), which verified the results of cell smear. The AMs in BALF accounted for about 90% of CD45^+^ cells, and CD11b^lo^CD11c^hi^ AMs increased on day 28 following BLM treatment, whereas HSM treatment reduced 35% of AM infiltration (Fig. 2C). In addition, macrophages were identified in the lung tissue. In contrast to BALF, CD11b^lo^CD11c^hi^ AMs and CD11b^hi^CD11c^lo^ IMs had very small proportions of CD45^+^ cells, which were around 4%. However, the numbers of AMs and IMs showed that HSM considerably blocked the accumulation of these tissue macrophages (Fig. 2D). Based on these results, we considered that HSM reduces infiltration of AMs into the BALF and inhibits their accumulation in the lung tissue.

### HSM inhibits autophagy of alveolar macrophages in fibrotic mice

**Figure 3:**
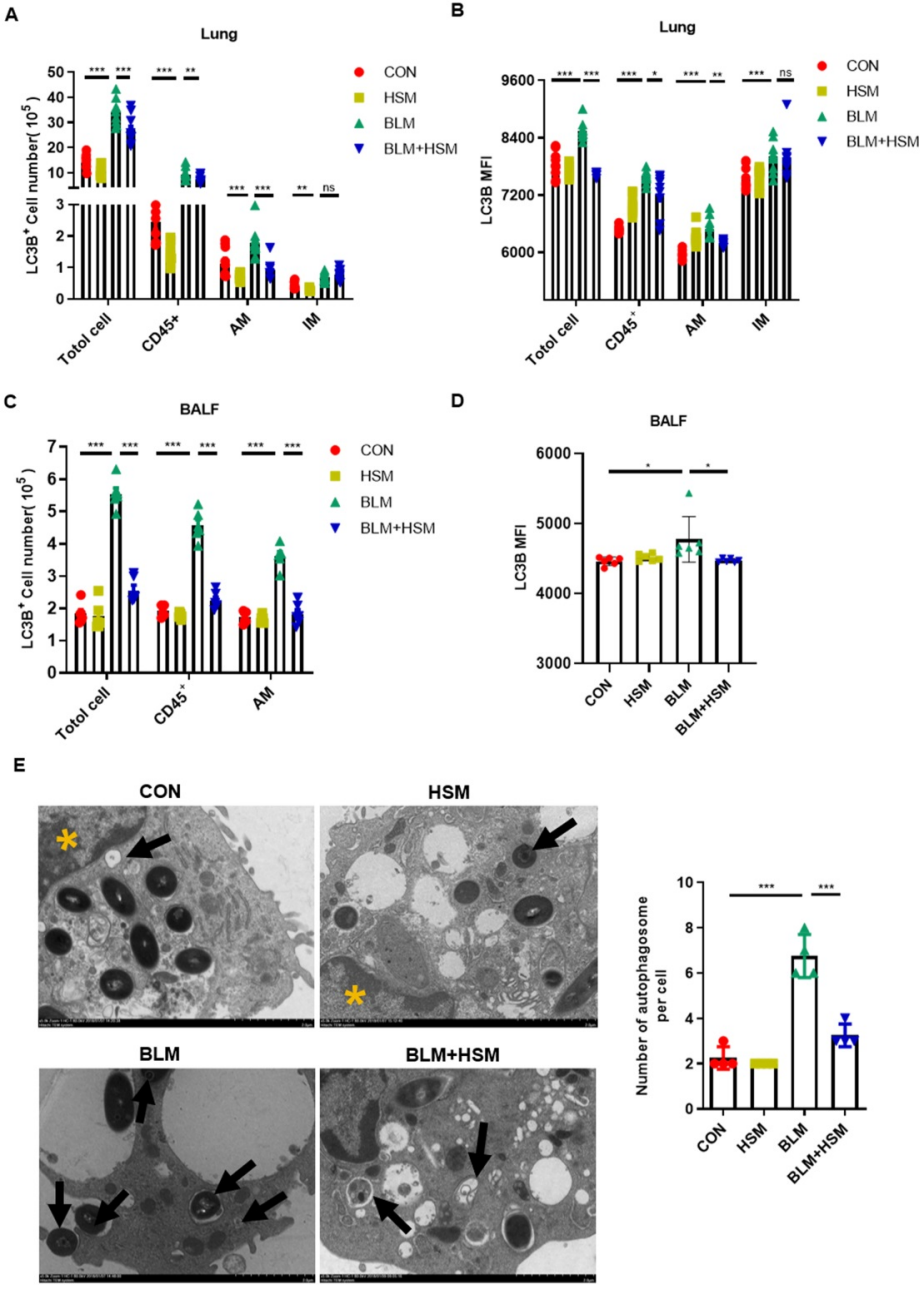
** HSM inhibits autophagy of alveolar macrophages in fibrotic mice.** (A) The effect of HSM on the count of LC3B positive cells (total cells, CD45^+^ leukocytes, alveolar macrophages and interstitial macrophages) in lung tissue, n=10. (B) The effect of HSM on LC3B MFI of total cells, CD45^+^ leukocytes, alveolar macrophages and interstitial macrophages in lung tissue, n=10. (C) The effect of HSM on the number of LC3B^+^ total cells, CD45^+^ leukocytes and alveolar macrophages in BALF, n=6. (D) The effect of HSM on LC3B MFI of alveolar macrophages in BALF, n=6. (E) Primary alveolar macrophages were extracted from BALF of each group of mice, and the changes of autophagosomes were observed by transmission electron microscopy, n=4. Black arrow: autophagosome; yellow asterisk: cell nucleus; scale bar: 2 μm. Data are presented as means ± SEM of at least three separate experiments. **P < 0.01, ***P < 0.001.

Excessive macrophage autophagy was thought to be related to pulmonary fibrosis 30. To evaluate whether the HSM effect on autophagy was associated with AMs, we monitored the LC3B protein level in leukocytes by flow cytometry. First, we observed that the total number of LC3B-expressing cells in lung tissue was significantly higher in the BLM model group compared with the saline-treated control group (Fig. 3A). In fibrotic lungs, BLM administration induced CD45^+^ leukocytes accounting for nearly 30% of the LC3B^+^ cells and these effects were significantly suppressed by HSM treatment (Fig. 3A). Furthermore, LC3B^+^ cells were elevated in AMs and IMs in the BLM group, which was reversed by HSM only in AMs, but not in IMs (Fig. 3A). Next, flow cytometry was used to evaluate the mean fluorescence intensity (MFI) of LC3B in lung tissue, MFI showed the same variation with the LC3B^+^ positive cell numbers (Fig. 3B). These data suggested that there was an inhibition effect of HSM on autophagy in AMs. Meanwhile, the change of LC3B expression was consistent with autophagy data of lung tissue in BALF, which was rich in over 90% AMs (Fig. 3C). Moreover, LC3B MFI in AMs from BALF samples in HSM-treatment group was further explained to be significantly downregulated compared with BLM model group (Fig. 3D). These results indicated that HSM inhibited LC3B expression in AMs in BLM-induced lung fibrosis, without affecting IMs.

In order to verify the effect of HSM on autophagy of AMs, we separated AMs from BALF of different treatment groups and examined the changes of autophagosomes by TEM (Fig. 3E). The number of autophagosome in AMs from the BLM model group were increased, and this effect was attenuated by treatment with HSM (Fig. 3E). Besides, studies have reported autophagy can maintain mitochondrial homeostasis and promote cell survival under oxidative stress 49, 50. JC-1 staining examined by flow cytometry was used to detect mitochondrial membrane potential (MMP) in AMs. The BLM model group had increased aggregated JC-1 in isolated AMs, HSM could promote more JC-1 monomer in AMs from fibrotic mice (Fig. S1A). These results suggested that HSM remarkably enhanced the collapse of the MMP. Bcl2 gene was identified to play a key role in cell survival and inhibition of apoptosis 51. Bcl2 staining indicated that there were reduced F4/80+ apoptotic macrophages in the model group, HSM reversed the inhibition of apoptotic cells in the lungs (Fig. S1B). JC-1 aggregates are suggestive of a resistance to apoptosis, and double staining of Bcl2 and F4/80 did prove HSM render alveolar macrophages less resistant to apoptosis. These results together reminded us HSM significantly decreased autophagy in the AMs of BLM-treated mice.

### HSM regulates autophagosome accumulation in macrophages dependent on TLR4 activity

**Figure 4:**
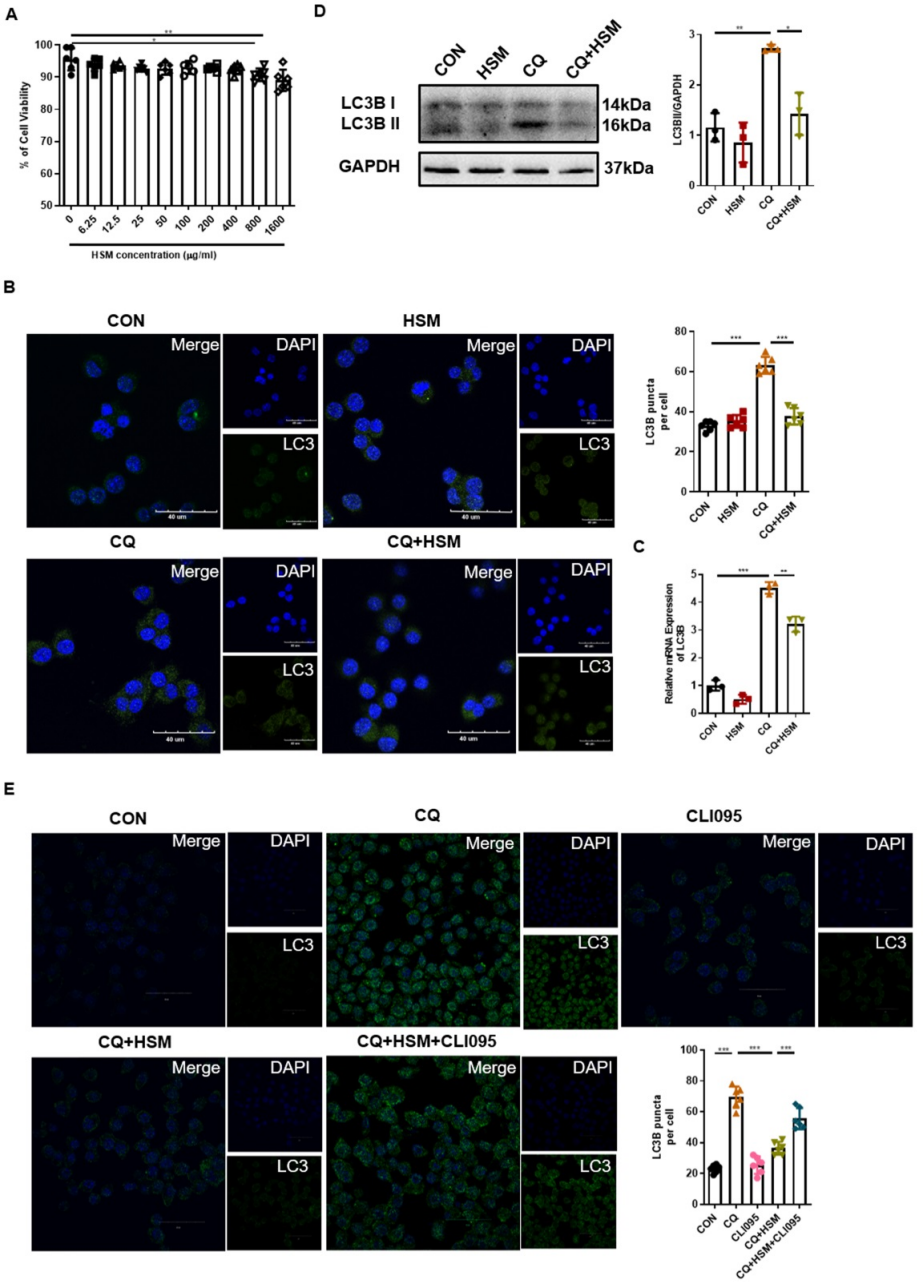
** HSM regulates autophagosomes accumulation in macrophages dependent on TLR4 activity.** (A) The effect of different concentrations HSM on the activity of RAW264.7 cells was analyzed by CCK-8 assay. (B) *In vitro* experiment to explore the effect of HSM on CQ-induced autophagy flux accumulation. RAW264.7 cells were pretreated with CQ (20 μM) for 2 h and then treated with or without HSM (16 μg/ml) for 16 h. Representative immunofluorescence image of LC3B in RAW264.7 cells treated with CQ and HSM. Scale bar: 40 μm. (C) The effect of HSM on the expression of *LC3B* mRNA was analyzed by q-PCR. (D) Western blotting to examine the effect of HSM on the expression of LC3B protein. (E) RAW264.7 cells were pretreated with CQ (20 μM) and CLI095 (100 μg/ml) for 2 h and then treated with or without HSM (16 μg/ml) for 16 h. Representative immunofluorescence image of LC3B in RAW264.7 cells treated with CLI095. Scale bar: 30 μm. Data are presented as means ± SEM of at least three separate experiments. *P < 0.05, **P < 0.01, ***P < 0.001.

After determining the effect of HSM on autophagy in AMs* in vivo*, RAW264.7 murine macrophage cells were used to study the autophagy regulatory effect of HSM* in vitro*. We first analyzed the cytotoxicity of HSM in RAW264.7 cells by CCK-8 assay. The results showed that HSM did not affect the cell viability at concentrations less than 400 μg/ml (Fig. 4A). Thus, we chose 16 μg/ml (HSM1) and 160 μg/ml (HSM2) as the final working concentration for the following experiments.

Abnormal autophagy activity as well as a block of autophagosome-lysosome can induce the accumulation of autophagosomes 52. CQ inhibits the binding of autophagosomes to lysosomes by increasing the pH of lysosomes, thereby blocking the flux of autophagy, leading to the accumulation of LC3B protein and preventing autophagy from proceeding normally 53. Next, we pre-treated RAW264.7 cells with CQ followed by HSM exposure, and immunofluorescence was used to detect the endogenous LC3B-II protein (Fig. 4B). CQ significantly induced LC3B-positive punctate structures in the macrophages, and HSM treatment resulted in a significant decrease in the number of LC3B-containing puncta (Fig. 4B). The *LC3B* gene expression and processed LC3B-II were measured by q-PCR and western blot, respectively. HSM showed similar reduction in CQ-induced autophagosome accumulation (Fig. 4C, 4D). Toll-like receptor 4 (TLR4) was reported to mediate autophagy in the pathological process of pulmonary fibrosis 54. We checked whether the regulation of autophagy by HSM was related to TLR4 activity. CLI095, also known as TAK-242, selectively inhibits signaling mediated by the intracellular domain of TLR4 55. Interestingly, CLI095 could block the effect of HSM on the accumulation of autophagosomes in the RAW cells (Fig. 4E). Thus, the above results indicated that HSM can reduce the accumulation of autophagosomes dependent on TLR4 activity, which will help to restore autophagy.

### Decreased TLR4/NF-κB signaling in macrophages with HSM incubation

**Figure 5:**
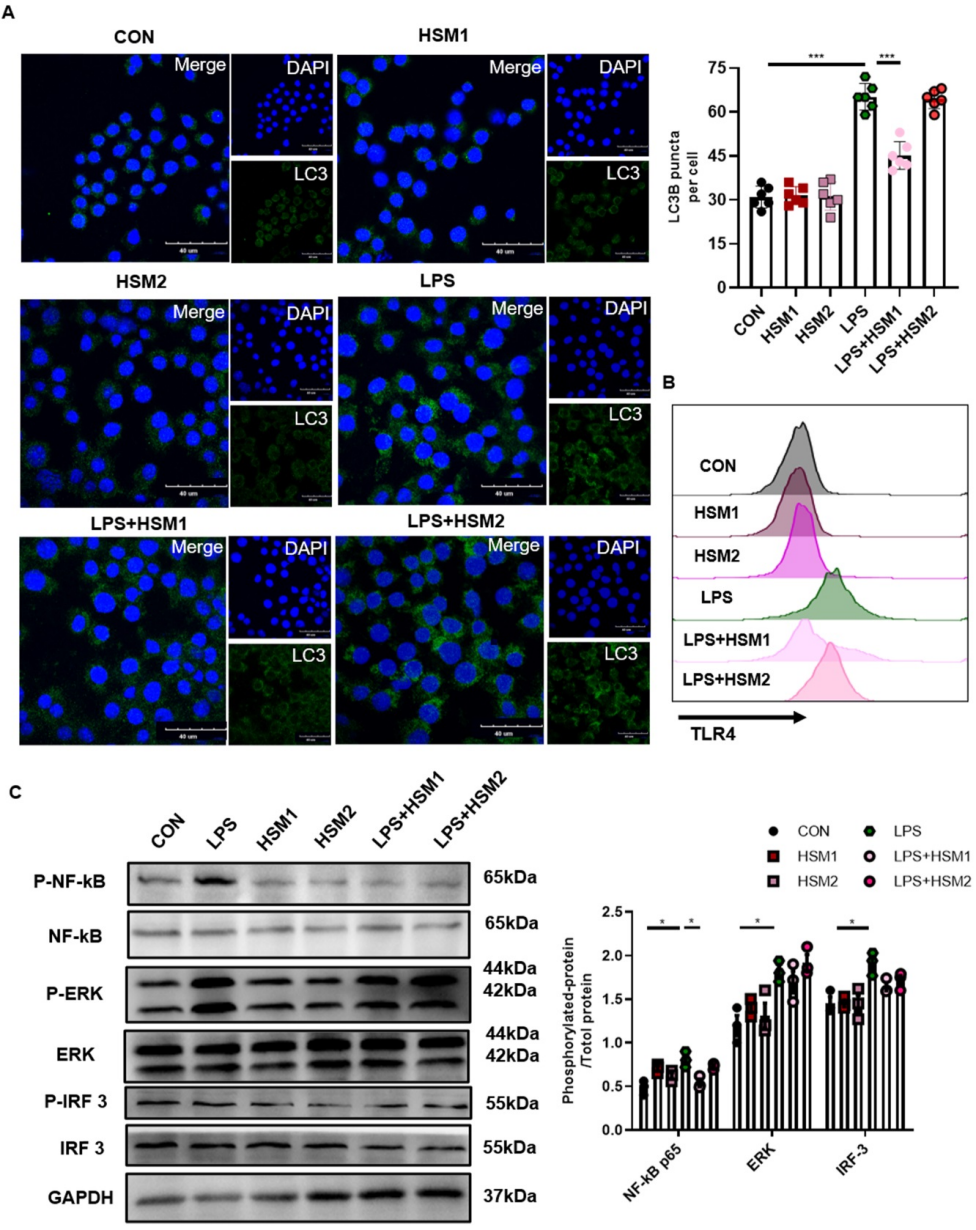
** Decreased TLR4/NF-κB signaling in macrophages with HSM incubation.** (A) RAW264.7 cells were pretreated with LPS (0.1 μg/ml) for 2 h and then treated with or without HSM (HSM1:16 μg/ml and HSM2: 160 μg/ml) for 16 h. Representative immunofluorescence image of LC3B in RAW264.7 cells treated with LPS. Scale bar: 40 μm. (B) Effect of HSM on TLR4 protein expression by flow cytometry. (C) Effect of HSM on the expression of TLR4 downstream proteins NF-κB P65, ERK and IRF 3 was analyzed by western blotting. Data are presented as means ± SEM of at least three separate experiments. *P < 0.05, ***P < 0.001.

LPS activates NF-κB translocation through TLR4 in AMs, and stimulates TLR4 in primary human macrophages and RAW264.7 cells to activate autophagy 56, 57. To investigate the role of HSM on LPS-induced TLR4-associated autophagy in macrophages, we used LPS to activate TLR4 and then measured the expression of LC3B-related autophagosomes through immunofluorescence. In addition, we not only repeated the previous dose of HSM1 (16ug/ml), but also added the higher dose of HSM2 (160ug/ml) based on CCK-8 (Fig. 4A). Incubation of RAW cells with LPS led to the LC3B punctate staining, which is typical of autophagosomes (Fig. 5A). However, incubation of RAW264.7 cells with HSM1 after the LPS activation led to fewer autophagosomes, but HSM2 has no obvious effect (Fig. 5A). These data established the inhibition of abnormal autophagic response by HSM1 in murine macrophages after LPS stimulation.

We hypothesized that the contribution of HSM to macrophage autophagy might be due to the regulation of TLR4 expression and its downstream signaling pathways. TLR4 protein expression was detected by flow cytometry. LPS-induced TLR4 expression was markedly inhibited in cells treated with HSM1 compared to cells induced with LPS only (Fig. 5B). Consistent with the effect on autophagy, HSM1 affected TLR4 expression rather than HSM2 (Fig. 5B). In addition, based on LPS-induced NF-κB, MAPK and IRF3 signaling pathways, HSM selectively abrogated the induction of phosphorylated NF-κB p65 (Fig. 5C). These results indicated that the effect of HSM1 on macrophage autophagy is mediated through the TLR4/NF-κB signaling pathway.

## Discussion

**Figure 6:**
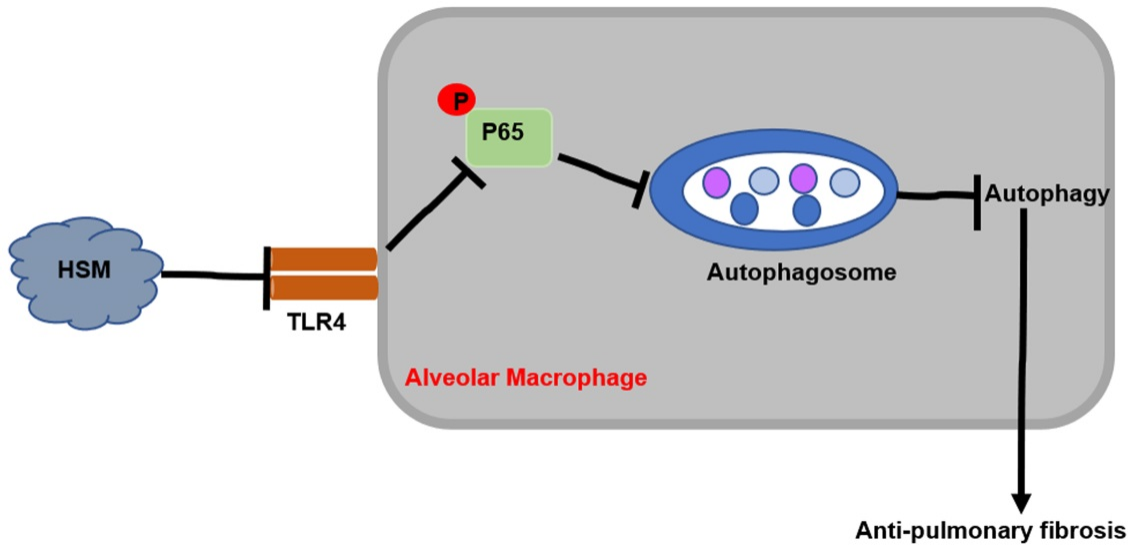
Mechanism of HSM treatment of BLM-induced pulmonary fibrosis in mice.

This study explored the potential protective effect of HSM on macrophages in attenuating BLM-induced pulmonary fibrosis. The results showed that HSM can inhibit excessive autophagic activity of AMs to achieve anti-fibrotic effect.* In vitro* studies showed that the effect of HSM on macrophage autophagy is mediated through the TLR4/NF-κB signaling pathway. These data suggested that AM autophagy might be a potentially novel and previously unrecognized target of HSM to limit pulmonary fibrosis, which had no effective therapies (Fig. 6).

Several studies have shown that pulmonary fibrosis is also accompanied by autophagy 35, 58-60, with an increase in the autophagic proteins. The level of microtubule associated protein LC3B, a unique molecular marker for autophagosomes, significantly increases in fibrotic lung tissues 35, 61. Yang *et al.* demonstrated that the conversion of LC3B-I to LC3B-II is increased in the BLM-injured lung tissues 54. A mouse autophagy RT-PCR array and protein level detection revealed that BLM-induced lung injury results in the up-regulation of ATG5 in lung tissue extracts 35. This study showed that HSM reduced autophagy activation by increasing LC3B-II and ATG5, and decreasing P62 protein levels (Fig. 1C). Consistent with these results, a decrease in autophagosome formation was also revealed (Fig. 1B). Thus, we preliminarily considered that the anti-fibrotic effects of HSM were associated with the regulation of autophagic activity. Autophagy is a normal physiological process in the body to maintain homeostasis and survival. It is reported that autophagy plays different roles in the pathogenesis of pulmonary fibrosis. Atg4b-deficient mice displayed more extensive and severe fibrosis with increased collagen accumulation 35. Impaired conventional ATG7-dependent macro-autophagy in lung endothelium increased susceptibility to BLM-induced fibrosis in mice 62. However, some studies suggested that autophagy may cause harm and accelerate disease progression. *In vivo*, autophagy is activated in fibrotic patients 36. There is widespread activation of autophagy in the lungs and pulmonary epithelial cells in the BLM-induced model 35. This autophagy induction phenomenon also appears in the fibrosis model caused by amiodarone 59. Additionally, the autophagy inhibitor 3-methyladenine reduced BLM-induced lung fibrosis 36.

Whether autophagy has a negative effect on human disease remains controversial, however, several studies have shown that macrophage autophagy may play a role in promoting fibrogenic diseases. IPF AMs show increased mitophagy and apoptosis resistance 28. Autophagy of AMs has been reported to regulate silica-induced pulmonary fibrosis 30, 63, 64. Our previous work and Fig. 2 demonstrated the anti-fibrotic effects of HSM associated with the regulation of AMs 11. The current study showed that HSM inhibited autophagosome number and LC3B expression level in CD11b^lo^CD11c^hi^ AMs without affecting IMs (Fig. 3A and B). And we verified this result by detecting the number of LC3B^+^ AM cells and the expression of LC3B MFI in BALF (Fig. 3C and D). This might indicate a role of AM autophagy in the anti-fibrosis mechanism of HSM.

Pulmonary macrophages may be used as targets for the treatment of pulmonary fibrosis due to their complexity and products in the process of fibrosis 65. Depletion of pulmonary macrophages slows the regression of fibrosis during BLM-induced pulmonary fibrosis 24. AMs are tissue-resident cells that play a primary role in maintaining immunological homoeostasis and host defense in lungs. Necrosis of monocyte-derived AMs can alleviate pulmonary fibrosis 66. Moreover, AMs can also participate in the process of pulmonary fibrosis by producing TGF-β 28. Engulfment of dysfunctional mitochondria by autophagosomes was reported in IPF AMs as well as apoptosis resistance 28. The occurrence of autophagy is essential for maintaining the homeostasis of mitochondria. The autophagy inducer rapamycin can enhance the red fluorescence intensity under JC-1 staining and maintain the normal function of mitochondria 50. Similarly, when HSM inhibited AM autophagy, MMP decreased and green fluorescence expression increased (Fig. S1A). Apoptosis and autophagy are two self-destructive processes in the body. Some research has indicated autophagy can regulate the occurrence of apoptosis. Autophagy is enhanced and conducive to cell adaptation and survival in various situations such as, the lack of growth factors and/or nutrition and hypoxia. Autophagy can inhibit apoptosis by removing pro-apoptotic proteins or adding caspase inhibitors 67, 68. HSM promoted the apoptosis of F4/80+ macrophages in the lung under BLM treatment (Fig. S1B). These results further suggested the inhibitory effect of HSM on autophagy of alveolar macrophages.

In our study, we found low-dose of HSM (16ug/mL, HSM1) could decrease the LC3B punctate staining. Nevertheless, when the concentration of HSM rose to 160ug/mL (HSM2), the inhibitory effect on LC3B did not maintain (Fig. 5A). Similarly, HSM2 rather than HSM1 did not affect TLR4 expression on the macrophages and the activation of downstream signaling pathways (Fig. 5B and C). Such opposite effects between high and low doses of treatment have been previously reported. Welshons wrote in the journal PNAS that diethylstilbestrol doses of 0.02, 0.2, and 2.0 ng per g of body weight per day increased adult prostate weight, whereas a 200-ng-per-g dose decreased adult prostate weight in male offspring 69. Tian et al. found that IL-2 high dose pretreatment ameliorated Con A-induced liver injury, while low dosage of IL-2 aggravated Con A-induced liver injury 70. Similarly, effects of low and high doses of Abeta42 on electrical network and neuronal excitability in the rat prefrontal cortex were opposite 71. In our study, such specific reasons underlying this phenomenon still need to be explored further. And the data strongly suggest that HSM may reduce the expression of LC3B and TLR4 signaling within a certain range of doses.

LPS-induced mTOR phosphorylation was down-regulated when TLR4 was knocked down, indicating that mTOR activation is caused by the TLR4 signaling pathway 72. When the TLR4 signaling pathway is stimulated, MyD88 and TRIF can be recruited simultaneously to mediate the release of pro-inflammatory cytokines 73. Our previous research found that HSM treatment of pulmonary fibrosis would cause the inhibition of mTOR activation and accompanied by inflammation suppression 11, 13. These results suggest that TLR4 signaling may be related to the efficacy of HSM. Our results showed that HSM did exert its autophagy inhibition effect through TLR4 signaling (Fig. 4E). In addition, we also analyzed the changes in several other pathways downstream of TLR4. As downstream signaling pathways of TLR4, MAPK family, NF-κB and IRF3 have been reported to regulate cell growth and inflammation, leading to pulmonary fibrosis 74-76. We found that HSM inhibited the activity of NF-κB but not the MAPK/ERK or IRF 3 pathway (Fig 5C). Consistent with this result, it has been reported that the inhibition of NF-κB signaling reduces the incidence of IPF *in vivo*, and reduces the proliferation of myofibroblasts and epithelial-mesenchymal transition *in vitro* 77-79. These results indicated that HSM played an important role via the TLR4/NF-κB pathway.

In summary, the results of this study suggested that HSM exerts anti-fibrotic effects through the inhibition of autophagy in AMs, and the underlying mechanism is related to the inhibition of the TLR4/NF-κB p65 pathway. Hence, HSM may be a potential therapeutic candidate for the treatment of pulmonary fibrosis, and should be investigated in future studies.

## Supplementary Material

Supplementary information.

## Abbreviations

AM: alveolar macrophage
BALF: bronchoalveolar lavage fluid
BLM: bleomycin
CQ: Chloroquine
HSM: *Hirsutella sinensis* mycelium
IM: interstitial macrophage
IPF: Idiopathic pulmonary fibrosis
LC3B: Light Chain 3
LPS: lipopolysaccharide
MFI: mean fluorescence intensity
MMP: mitochondrial membrane potential
SPF: specific pathogen-free
TEM: transmission electron microscopy
TGF-β: transforming growth factor-β
TLR4: toll-like receptor 4
