# Supplementary material for: Hirsutella sinensis mycelium regulates autophagy of alveolar macrophages via TLR4/NF-κB signaling pathway
Source: Int J Med Sci. 2021 Feb 18;18(8):1810–23. doi: 10.7150/ijms.51654 (PMC7976595; doi:10.7150/ijms.51654)
Supplement: Supplementary file 1 — Supplementary information. [file ijmsv18p1810s1.pdf]

Mitochondria Membrane Potential

Mitochondrial membrane potential (MMP) in control and treated group of mice was determined by JC-1 (Fcmacs, Nanjing, China). In functional mitochondria, JC-1 spontaneously forms complexes known as J-aggregates that produce intense and discrete red fluorescence. In the mitochondria of abnormally functioning apoptotic or unhealthy cells, JC-1 remains in the monomeric form that emits bright green fluorescence [1, 2]. BALF was treated with JC-1 at 37 °C for 30 min, and unbound JC-1 was pipetted with PBS buffer. The fluorescence of JC-1 was detected by flow cytometry.

Immunofluorescence

To assess HSM treatment mediated survival of macrophages that are present in lung tissues, we performed an apoptosis assay on mice lung tissue. The antibodies used for Immunofluorescence detection were F4/80 Polyclonal Antibody (Proteintech, Rosemont, IL, USA) and BCL2 Monoclonal antibody (Proteintech, Rosemont, IL, USA). The secondary antibodies used were FITC-goat anti-Rabbit IgG (H+L) and Cy3-AffiniPure goat Anti-Mouse IgG (H+L) (Jackson ImmunoResearch, Pennsylvania, USA). DAPI (Fcmacs, Nanjing, China) was used for nuclear counterstaining. Cells were imaged on a FV10i confocal microscope (OLYMPUS, Japan), and Image J was applied for data analysis.

A

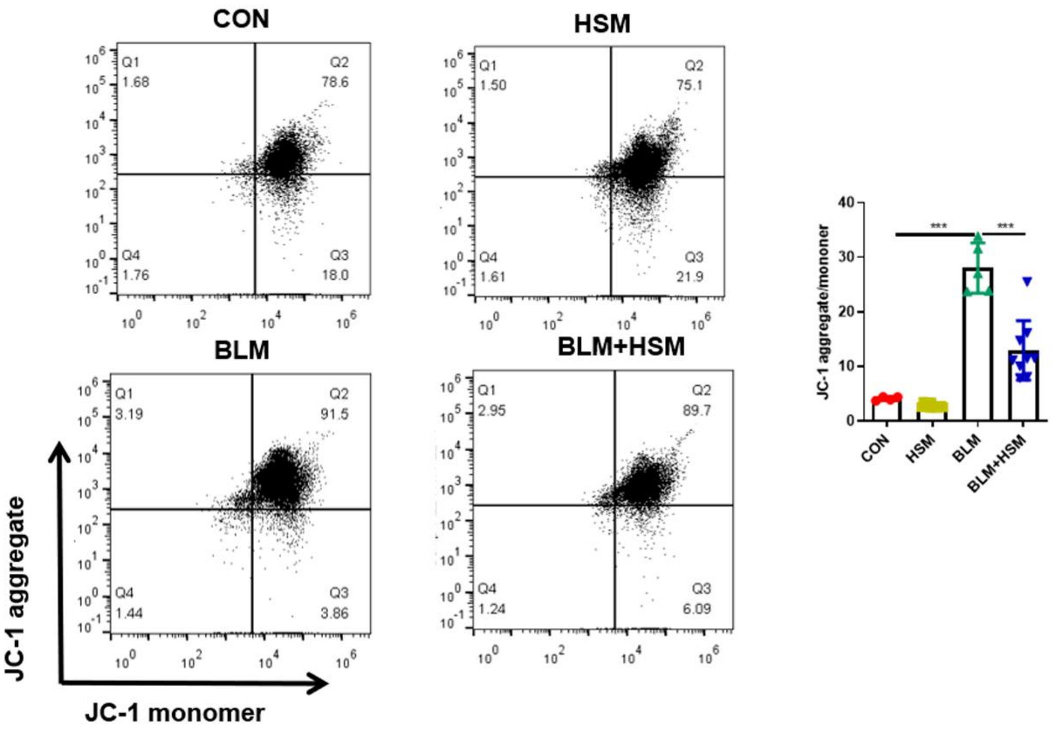

B

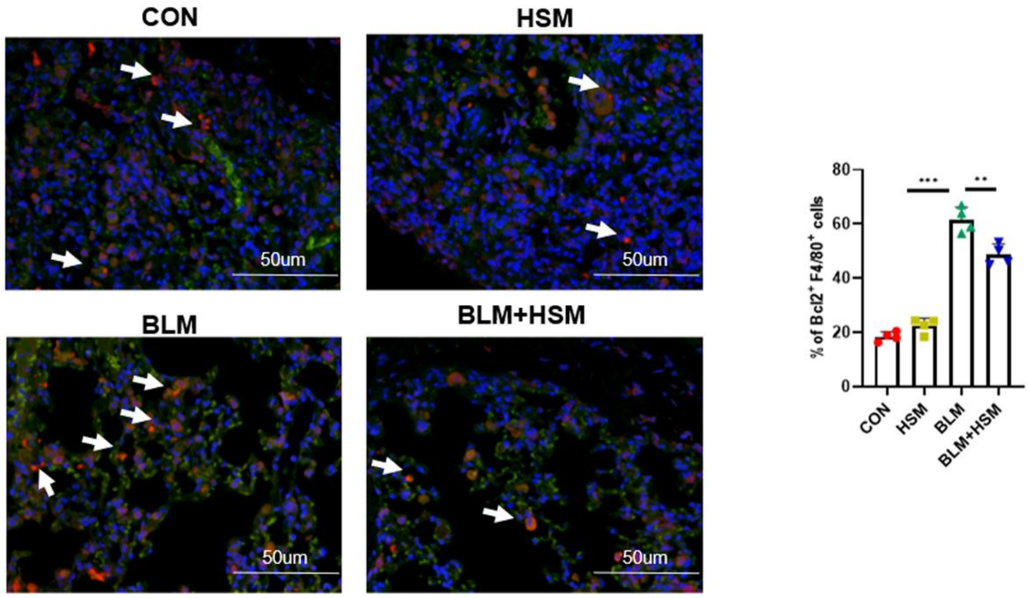

Figure S1. Changed mitochondrial membrane potential and apoptosis by HSM in mice with pulmonary fibrosis (A). Examining changes in mitochondrial membrane potential in BALF of pulmonary fibrosis mice, n=4. (B). Representative immunofluorescence image of F4/80 and Bcl2 double staining were presented. Cell death was measured by Bcl2 (red) staining, and macrophages

were identified by the macrophage surface marker, F4/80 (green), n=4. Scale bar: 50 μm. Data are presented as means ± SEM of at least three separate experiments. $^{**}P < 0.01$, $^{***}P < 0.001$.

References

1. Smiley ST, Reers M, Mottola-Hartshorn C, et al. Intracellular heterogeneity in mitochondrial membrane potentials revealed by a J-aggregate-forming lipophilic cation JC-1. Proc Natl Acad Sci U S A. 1991; 88: 3671-5.
2. Akhtar MJ, Ahamed M, Alhadlaq HA, et al. Mitochondrial dysfunction, autophagy stimulation and non-apoptotic cell death caused by nitric oxide-inducing Pt-coated Au nanoparticle in human lung carcinoma cells. Biochim Biophys Acta Gen Subj. 2020; 1864: 129452.
